# Supplementary material for: Effective resistance against pandemics: Mobility network sparsification for high-fidelity epidemic simulations
Source: PLoS Comput Biol. 2022 Nov 22;18(11):e1010650. doi: 10.1371/journal.pcbi.1010650 (PMC9681106; doi:10.1371/journal.pcbi.1010650)
Supplement: Attachment — Submitted filename: AM_SS_CM_PLOSCompBio_RevResponse.pdf [file pcbi.1010650.s001.pdf]

July 13, 2022

To the Editors of PloS Computational Biology,

This letter is in reply to reviewer comments sent to us on June 10, 2022. Reviewer comments are in black while our replies are in blue. Enumeration of reviewer comments was added for additional ease in reading. We thank both reviewers for their careful reading of the draft, and we found their comments very helpful.

Reviewer One Comments:

1. In the introduction, the authors stated, "It outperforms the simpler edge-sampling methods based on uniform probabilities and edge weights, as well as the naïve thresholding approach." Simply saying "outperform" is a little vague. I think it would be better if the authors could explain precisely how the effective resistance sampling method is better than the other methods. It seems that the effective resistance sampling method is more accurate to capture the behavior of the SIR model, but it is more computationally expensive than the other simpler sparsification algorithms.

   We agree that the blanket verb "outperforms" is vague. Accordingly, we have changed the sentence to be more specific about the metrics we use throughout the paper and also provide a clarification about the computational cost. This now reads as follows:

   "Here we confirm on a dense real-world mobility network that the Spielman–Srivastava algorithm preserves the behavior of the SIR model, even when we keep only a few percent of the original edges. This includes both the bulk behavior of the epidemic and the probability distribution of events, including the probability that each node becomes infected and the distribution of times at which it does so. According to these metrics, it achieves higher fidelity than simpler edge-sampling methods where the probability is uniform or proportional to edge weight, as well as the naive thresholding approach. While estimating the effective resistance for each edge has a higher computational cost than these methods, this cost is only incurred once for each network as a preprocessing step, after which independent trials of the SIR simulation (and, if desired, of the edge-sampling process) can be carried out at no additional cost."

2. Figure 1: Is the color coded on the same scale for the figures on the left and right? The edges look in the same darker blue in the figure on the left.

   The edges are color coded on a log scale such that darker blue edges are lower edge weight and lighter blue edges are higher edge weight, with both the left and right side of the figure following the same scale. This was done to illustrate how the Spielman–Srivastava algorithm re-weights edges to compensate for sparsification. The Figure 1 caption has been modified to make this clearer.

3. It would be better if the authors could provide some details of the network in the caption of Figure 1. For example, each node represents a census tract, define q as the fraction of edges wanted to preserve, etc.

   The caption of Figure 1 has been expanded to encompass these details. Along with addressing the reviewer's previous question, the caption now reads as follows:

   The U.S. mobility network, where nodes are census tracts and edges are weighted according to average human mobility between census tracts. On the left, the original network with about 26 million edges. On the right, a sparsified network based on effective resistance sampling with $q = 0.1$, preserving about 7% of the edges of the original. Heavier-weight edges are lighter in color on a logarithmic scale. Note the mixture of local and long-range links, and how sparsification reweights edges to heavier in order to compensate for the decrease in density.

4. Figure 2: There are blue and black dots in the figure. What does the color represent?

   The blue dots in Figure 2 show the 90% of points closest to the diagonal in order to show the spread and density of the scatter plot in relation to the diagonal. The caption of Figure 2 has been changed to reflect this; see response to next comment.

5. Moreover, weight-based sampling method performed almost as good as the effective-resistance method, at least when measured by R-squared. I think the authors should mention this observation in the text.

   We have updated the caption of Figure 2 to reflect the similarity in R^2, while also pointing out the differences in the infection probability for low-probability nodes. This caption now reads as follows:

   "Dots close to the diagonal are those for which sparsification preserves this probability, and the blue dots represent the 90% of nodes closest to the diagonal. While weight-based sampling achieves a similar R^2, it is not as good at preserving

infection probabilities for low-probability nodes, especially for dispersed initial conditions. Effective resistance preserves the infection probability for both low- and high-probability nodes."

6. The authors chose the same q values for the three edge-sampling methods and simulated the methods for each value of q. So in Figures 3 and 5, if I draw a vertical line on each chosen value of q, should three different dots lie on the vertical line? It seems that the dots are aligned for q values below 0.01 but not for the values above 0.01.

   The x-axis for Figures 3 and 5 is not q (the number of edge samples divided by m) but the fraction of distinct edges included in the sparsified network. As discussed in the Introduction, edge-sampling techniques can choose the same edge multiple times, in which case this fraction is typically less than q. For instance, in the text associated with Figures 1 and 2, we state that $q = 0.1$, but that the fraction of distinct edges preserved is 7% or 0.07. Moreover, sampling according to a non-uniform distribution, such as one proportional to edge weight or effective resistance, is more likely to produce these repeated samples, shifting x slightly to the left compared to uniform sampling. This effect is lessened at smaller q, since then it is less likely for edges to be chosen multiple times.

   To make this clearer, we have added the following to the caption of Figure 3: "The horizontal axis shows the fraction of distinct edges of the original network preserved by the sparsifier. Note that this fraction is slightly less than q, since edge-sampling algorithms can choose the same edge multiple times."

7. Shaded regions corresponds to one standard deviation of the average." However, I didn't see the shaded regions in the figure on my end. Also, "corresponds" should be "correspond".

   The shaded regions in Figure 3 are present. However, the shaded region is relatively small, especially for (A) of Figure 3. We have updated the caption to read "The shaded regions correspond to one standard deviation of the average, which for several of these curves is too small to see."

8. I don't understand Figure 4 very easily. Please provide a better description. What does the y-axis represent? How are these two nodes chosen? Is it meaningful that all sampling methods have overlapping curves for one node under the localized initial condition but not the dispersed initial condition?

   The y-axis is the probability density of the time at which a node becomes infected; since this is a probability density function, the area under each curve is 1. For instance, on the upper left the typical arrival times range over an interval of width about 1/6, giving a probability density that peaks at 6. Each of the four graphs shows this distribution for a particular node and a particular initial condition. We have added a label to the y-axes ("probability density") and expanded the caption as follows, which we hope is clearer:

   "Arrival time distributions for the original network (Org) and sparsified networks produced by the three edge-sampling methods with 7% of the original edges preserved. In each graph, we show the probability density of the time at which a particular node becomes infected, conditioned on the event that it becomes infected during the epidemic. We show this distribution for two representative nodes (top and bottom) under (A) the localized initial condition and (B) the dispersed initial condition. The top node is in a well-connected part of the network, with typical arrival times ranging from 0.5 to 1.8 in the localized initial condition and from 0.05 to 0.25 in the dispersed initial condition. The bottom node is in a sparser region and more remote from the initial infection, giving it arrival times of 3–8 and 0.2–1.5 in the localized and dispersed initial conditions respectively. All three edge-sampling methods do fairly well at reproducing the shape of these arrival time distributions."

Reviewer Two Comments:

1. While the effective resistance methods is statistically-principled ("the weighted adjacency matrix and graph Laplacian of $\tilde{G}$ are equal, in expectation, to those of G"), distance backbones are algebraically-principled (and parameter-free). Indeed, distance backbones are unique for a given distance function (typically in network science we sum distance edges resulting in the unique metric backbone, but other distances are possible), while effective resistance leads to different sparsifications in each run (similarly to the disparity filter proposed by Serrano, Boguna and Vespignani [Ref. 15] but likely more efficient in preserving spreading dynamics given the preservation of essential connectivity), and also changes the original edge weights.

   This is an excellent point. To improve our discussion of the backbone vs. effective resistance, we have deleted the sentence "But we know of no rigorous results on whether these techniques preserve dynamical behavior" and replaced it with the following:

"In particular, the distance backbone defined in [SimasCorreiaRocha] preserves all shortest-path distances on a weighted graph. This is clearly important to many types of dynamical systems on a network. On the other hand, epidemic spread is a setting in which many parallel paths can combine to transmit a disease more quickly than a single shortest path. Our goal is to sparsify networks in a way that takes this effect into account."

We think this addresses how the goals of effective resistance differ from that of the distance backbone, and why resistance might be the right quantity to consider for epidemic spread. But we agree with you that it makes sense to explore this further; see below.

2. It would be interesting to understand how the effective resistance sparsification affects the original distribution of shortest paths? Figure 4 tallies the fraction of disconnected nodes, which is related, but not the distribution of shortest paths. This suggests that effective resistance preserves the distance backbone for a large range of the fraction of edges removed (unlike the other methods) as the backbone would also keep all edges connected, but the impact on shortest paths may occur for smaller fractions of edges removed. So, while the effective resistance "sparsifier preserves the linear properties of the original networks in expectation," the distance backbone does not affect any shortest path on the network (nor the original distance weights). This speaks to the synergy between the two concepts, and of course I do not expect the authors to run simulations to compare the two methods in this paper. However, the impact of effective resistance sparsification on the distribution of shortest paths is a reasonable question to consider—in addition to the uniqueness of the distance backbone.

   The preservation of the distribution of shortest path lengths by effective resistance sampling is an interesting question. While reweighting edges will often change the distance between their endpoints, the overall distribution (over all pairs of nodes) may stay roughly the same. We agree with the reviewer that this is an interesting direction for future work.

3. Another related question is what to do with the results of the effective resistance sparsifier since it changes the original weights? In the case of the epidemic models this is (more or less) clear because we tend to be more interested in the dynamical observables reported (time to infection, infection probability, etc.) But what about cases where the original weights are meaningful? For instance, in brain connectome or gene regulation networks (as in [Ref. 16]) the original edge weights have specific experimental significance. The distance backbone preserves the edge weights and thus their experimental significance. The authors could discuss or suggest how that would be handled with their sparsifier.

   This is a good point. In our setting, the goal of sparsification is to reduce the number of edges while preserving epidemic dynamics. To do this, edge-sampling algorithms reweight edges to maintain the overall conductance of the network. For instance, if there are many low-weight edges crossing between two communities, the algorithm will choose one of these edges and give it a large weight, essentially asking it to stand in for the entire bundle of parallel edges. While this may do a good job of preserving dynamics, it certainly obscures the original weights, which as you say have scientific significance.

   To address this, we have added the following sentence to the Conclusion:

   "One caveat is that, while this method of sparsification preserves epidemic dynamics, it can obscure the original edge weights. For instance, if there is a bundle of low-weight edges that cross between two communities, the Spielman–Srivastava algorithm will choose one of them and give it a high weight, in essence designating it as the representative of the entire bundle. This makes sense in contexts like epidemic spreading where bundles of parallel edges can work together and act as one high-weight edge. But in some other contexts such as genetic regulatory networks, where the goal is to understand the functional role of each link and where edge weights are of independent scientific interest, weight-preserving methods like those of [SimasCorreiaRocha,Gates-etal] may be more appropriate."

4. The authors say, in regards to Refs 16&17, that they "know of no rigorous results on whether these techniques preserve dynamical behavior." The effective graph methodology [Ref. 17] is also principled (logically-principled based on the Quine-McCluskey algorithm) , but it applies to networks with node dynamics (such as automata networks used in systems biology). The effective graph is unique because our measure of effective connectivity is a parameter (not a statistic) of the dynamics of Boolean functions. Preserving dynamical behavior is the whole point of Ref. 17, indeed of the whole approach. Because the networks analyzed in this paper have no node-dynamics and are rather used to study spreading dynamics (dynamics on networks rather than dynamics of networks), the effective graph is not directly comparable to the effective resistance methodology (albeit the similar name). Still, one cannot say the latter was not studied in regards to preserving dynamical behavior (our recent discussion in https://doi.org/10.1093/bioinformatics/btac360 could be relevant here).

We agree with you—we should have read this paper more carefully—and we have deleted that sentence (see response to comment #1 above).

5. As for whether the distance backbone methodology [Ref. 16] was studied to preserve dynamical behavior (in the sense of dynamics on networks) is a curious thing since https://doi.org/10.1101/2022.02.02.478784 is under review in this same journal, and of course the authors would not know about it. Still, the utility of the distance backbone in epidemic models has also been studied. I would venture that the effective resistance sparsifier assumes propagation by a particular distance (a resistance distance) which makes a lot of sense for epidemic spread, whereas the distance backbone methodology at large can consider any distance (our under review work on epidemic spread considers only the metric distance). I posit that it should be possible to derive a "resistance backbone" that is unique (not sampled) , but that is clearly an idea for future work—again, supporting the synergy and complementarity between the two methods, which in my view are both very relevant and useful.

   Your idea of combining the two approaches is intriguing. One difficulty is that every edge has at least a small effect on the effective resistances, not just between its own endpoints but between any two nodes for which some path (not necessarily the shortest one) passes through that edge. So unlike the shortest-path distance, no edge can simply be removed.

   On the other hand, one could remove edges whose effect on effective resistances is below some threshold. This might lead to an alternate strategy for Laplacian sparsification, which would also be of broad interest in computer science. Note that there are also deterministic variants of the Spielman-Srivastava algorithm, which use a greedy strategy to decide which edge to include.

6. I would welcome a little more justification for the values of the edge-sampling proportion q used. In particular, why only 0.1, 0.55, and 1 for anything above 10%? Probably because there is not much difference between methods tested (safe thresholding) in that range? By the way, since other figures are derived for q=0.1 which led to near 7% edges preserved (right?), a figure with q vs actual % od edges that remain would be useful even if in supporting materials as I imagine this may different from network to network depending on how many (essential) bridges they have (and other properties).

   Your intuition is correct: the behavior of all three edge-sampling methods is fairly good when q is large. But since our goal is to sparsify the network as much as possible, we focused on performance at smaller q, and found that effective resistance preserves dynamics with greater fidelity than other methods at these densities. To put it differently, effective resistance is better at compressing the network while maintaining epidemic dynamics.

   As you say, the number of distinct edges in a sparsifier is less than or equal to the number of samples, since edge-sampling algorithms can choose the same edge multiple times. This effect is greatest for nonuniform distributions (including weight and effective resistance) and at larger q. For these distributions, it is also network-dependent, since it depends on the distribution of weights (and for effective resistance, the network topology).

   We include a figure below showing this relationship for the US mobility network. However, with the reviewer's and editor's permission, we would rather not create an additional supporting materials document: currently the paper is self-contained, and we discussed this effect in the Introduction. To address the reviewer's concerns, we have updated the caption of Figure 3 to include the following: "The horizontal axis shows the fraction of distinct edges of the original network preserved by the sparsifier. Note that this fraction is slightly less than q, since edge-sampling algorithms can choose the same edge multiple times."

7. I don't quite get what all the four panels are in Figure 4. A and B sides are explained, but not upper and lower panels.

   The other reviewer also found this figure confusing, and we agree. The upper and lower panels correspond to two representative nodes, for each of which we measure the arrival time distribution in the two initial conditions. We have modified the figure caption as follows:

   "Arrival time distributions for the original network (Org) and sparsified networks produced by the three edge-sampling methods with 7% of the original edges preserved. In each graph, we show the probability density of the time at which a particular node becomes infected, conditioned on the event that it becomes infected during the epidemic. We show this distribution for two representative nodes (top and bottom) under (A) the localized initial condition and (B) the dispersed initial condition. The top node is in a well-connected part of the network, with typical arrival times ranging from 0.5 to 1.8 in the localized initial condition and from 0.05 to 0.25 in the dispersed initial condition. The bottom node is in a sparser region and more remote from the initial infection, giving it arrival times of 3–8 and 0.2–1.5 in the

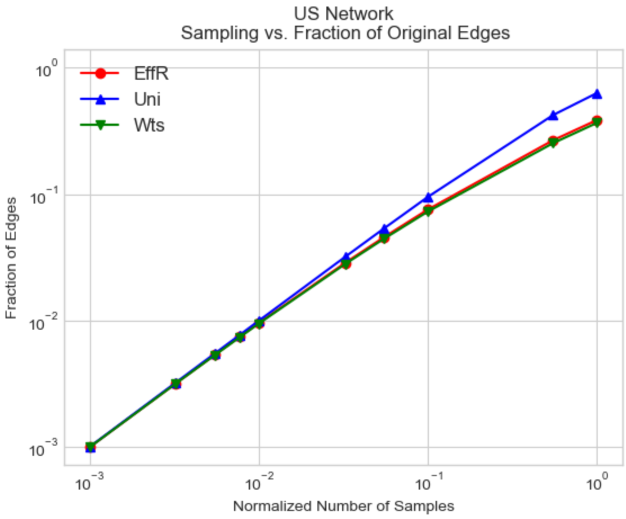

Figure 1: *The fraction of distinct edges included vs. the normalized number of edge samples $q = s/m$ for Uniform (Uni), Effective Resistance (EffR), and Weights (Wts) Sampling. This shows the effect discussed in the Introduction that the same edge can be sampled multiple times.*

> localized and dispersed initial conditions respectively. All three edge-sampling methods do fairly well at reproducing the shape of these arrival time distributions."

We are grateful to both referees for their comments, and we feel this revised paper is significantly clearer. Thank you for your consideration,

Alexander M. Mercier
Ph.D. Student
T.H. Chan School of Public Health
Harvard University
amercier@g.harvard.edu

Samuel Scarpino
Pandemic Prevention Institute
Health Initiative
The Rockefeller Foundation
sscarpino@rockfound.org

Cristopher Moore
Professor
Santa Fe Institute
moore@santafe.edu
